# Is superior tibiofibular joint resection necessary in extraarticular knee resection for sarcomas? A systematic review

**DOI:** 10.1186/s12957-016-0783-y

**Published:** 2016-02-03

**Authors:** Magdalena M. Gilg, Christine Wibmer, Dimosthenis Andreou, Patrick Sadoghi, Georg Gosheger, Andreas Leithner

**Affiliations:** 1 Department of Orthopedics and Orthopedic Surgery, Medical University of Graz, Auenbruggerplatz 5, 8036 Graz, Austria; 2 Department of General Orthopedics and Tumor Orthopedics, University Hospital of Muenster, Albert-Schweitzer-Campus 1, Gebäude A1, 48149 Münster, Germany

**Keywords:** Sarcoma, Superior tibiofibular joint, Extraarticular resection

## Abstract

**Background:**

Sarcomas infiltrating the knee joint require extraarticular resection to achieve wide margins. Opinions differ as to whether the superior tibiofibular joint (STFJ) is part of the knee joint and should be removed in the course of extraarticular resection. Thus, we investigated the frequency of communication between the tibiofemoral joint (TFJ) and the STFJ, and the reported local recurrence rates (LRR) following extraarticular knee resection.

**Methods:**

A systematic literature review on STFJ and TFJ communication and local recurrence rates following extraarticular knee resections was undertaken.

**Results:**

Cadaver studies detected communication between the TFJ and STFJ in 10–64 % of the cases. Direct arthrography with physical loading verified a 100 % communication rate. Regarding the extent of extraarticular knee resection, two institutions where the STFJ was resected had a LRR of 4–8 %, while studies from another three where the STFJ was not routinely resected reported a LRR of 0–21 %.

**Conclusions:**

Since the literature reports about a 100 % communication rate between the TFJ and the STFJ, resection of the STFJ in patients with sarcomas involving the knee joint would seem to be indicated, although it is not clear whether resection of the STFJ reduces local recurrence rates.

## Background

Surgical resection of the primary tumor with wide margins remains, with a few exceptions, the gold standard for local treatment of patients with soft tissue and bone sarcomas [1, 2]. The extent of resection varies depending on the extension of the tumor along anatomical structures or compartments. Only a small number of patients have sarcomas that involve the knee joint and require extraarticular knee resection [1]. This involvement can be the result of inappropriate placement of the biopsy tract, tumor extension along the cruciate ligaments, previous pathological fracture due to localization of a primary tumor within the joint, or direct infiltration of the joint [1, 3–5]. Surgical reconstruction techniques include modular tumor endoprotheses, muscle flaps, tendon transfers, allograft-prosthetic composites, or knee arthrodeses [3], all with varying functional outcomes [1]. Reconstruction of the extensor mechanism of the knee can be achieved either by splitting the tendons and patella during tumor resection or by removing the entire extensor apparatus followed by muscle flap or allograft reconstruction [3]. In contrast to intraarticular resection, complication rates and secondary amputation rates are reported to be higher in extraarticular knee resections [4]. Gosheger et al. [6] found a 6.2-fold higher risk of infection in extraarticular resection with distal femoral replacement than with intraarticular resection (*p* = 0.004).

Anatomically, the knee joint is composed of the tibiofemoral joint (TFJ) and the femoropatellar joint [7]. The superior tibiofibular joint (STFJ) is in close proximity to the knee joint. According to anatomy textbooks, it is not considered to be a part of the knee joint, since there is no connecting capsule or communication between the compartments [7–9]. However, several anatomical studies have reported communication between the TFJ and STFJ after histopathological dissection of the knee joint [10–13]. The exact extent of sarcoma resection is of particular relevance for orthopedic tumor surgery and sarcoma management since inadequate resection margins result in high local recurrence rates [1, 14, 15].

To clarify whether the STFJ is to be removed when an extraarticular knee joint resection is performed, we conducted a systematic literature review about the frequency of communication between the TFJ and the STFJ as well as about reported local recurrence rates (LRR) following extraarticular knee resections for sarcomas.

## Methods

**Fig 1.**
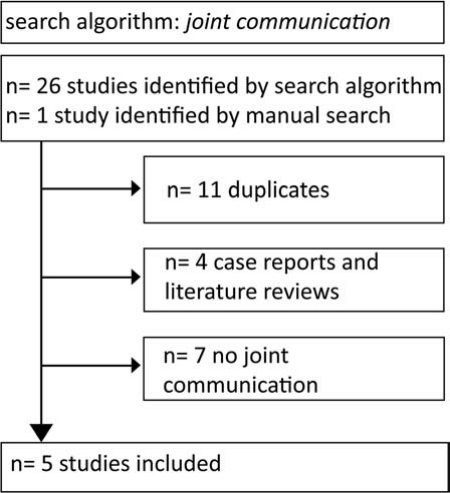
This shows the trial flow of study identification for the search algorithm of joint communication

**Fig 2.**
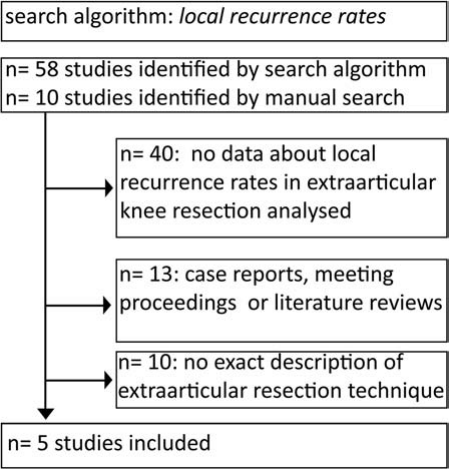
This shows the trial flow of study identification for the search algorithm of local recurrence rates

To answer our two research questions, two separate systematic literature reviews were performed, searching MEDLINE (via PubMed) and EMBASE (via OVID) for relevant studies published up to January 2014. The search algorithm for joint communication was “communication AND tibiofibular AND (knee OR tibiofemoral)”. Of 26 studies retrieved, 11 were duplicates, four were case reports or literature reviews, and seven did not contain data about joint communication. Reviewing bibliographies and manual literature research revealed one additional study. Finally, five studies could be included in our analysis (see Fig. 1). For inclusion, communication rates between the superior tibiofibular joint and the knee joint had to be reported based either on (A) in vivo investigation or (B) histopathological dissection. Regarding local recurrence rates, the search algorithm “resection AND knee AND (extraarticular OR extra-articular)” was used. Fifty-eight studies plus ten studies identified by manual research (bibliographies and searching peer reviewed orthopedic journals for relevant articles) were identified (see Fig. 2). Five studies fulfilled the following inclusion criteria: (1) local recurrence rate data available either from the text or calculable from data included in the text unequivocally analyzed for extraarticular knee resection, (2) English language publication in a peer reviewed journal, and (3) description of the surgical technique used for extraarticular knee resection. Forty studies were excluded due to absence of local recurrence rates; 13 were case reports, meeting proceedings or literature reviews and in ten cases, the surgical technique was not described clearly or there was no separate analysis of extraarticular and intraarticular knee resection.

All studies included for the analysis of communication were anatomical studies on cadavers or surgical specimens; there was also one in vivo study with arthrography and weight bearing. Regarding the level of evidence for the studies analyzing LRR, no RCT or observational study was retrieved [16]. Three retrieved studies were retrospective analyses, and two were non-randomized prospective case series. Two orthopedic surgeons independently reviewed the quality of the retrieved studies (MMG, CW). Disagreements were resolved by consensus. A third author (AL) was consulted if necessary.

## Results

Reported rates of communication between the TFJ and the STFJ ranged from 10 to 100 % [10–13, 17]. The studies were published between 1999 and 2013 and included a mean number of 15 patients. Four studies [10–12, 17] were conducted on cadavers using histopathological dissection and/or MRI arthrography and demonstrated consistent communication between the TFJ and the STFJ in 10 to 64 % of cases (see Table 1). There was one in vivo study in which, following contrast medium injection plus weight bearing, delayed MRI or CT showed a 100 % communication rate [13] (see Table 1).

**Table 1:** This table shows the results of the literature review regarding communication rates between STFJ and TFJ

Author	Study title	Journal	Number of specimen	Type of specimen	Method	Frequency of communication (%)
Espregueira-Mendes J. D. et al. (2005) [12]	*Anatomy of the proximal tibiofibular joint*	Knee Surg Sports Traumatol Arthrosc	20	Autopsy	Histopathological dissection	10
Bozkurt M. et al. (2003) [10]	*The Proximal Tibiofibular Joint An Anatomic Study*	Clin Orthop Relat Res	14	Autopsy	MRI arthrography plus histopathological dissection	64
Puffer R. C. et al. (2013) [13]	*CT and MR Arthrograms Demonstrate a Consistent Communication Between the Tibiofemoral and Superior Tibiofibular Joints*	Clin Anat	17	In vivo	MRI/ CT arthrography plus weight bearing	100
Dirim B. et al. (2008) [11]	*Communication Between the Proximal Tibiofibular Joint and Knee via the Subpopliteal Recess: MR Arthrography with Histologic Correlation and Stratigraphic Dissection*	AJR	12	Autopsy	MRI arthrography plus histopathological dissection	27.5
Hyman J. et al. (1999) [17]	*Anatomy Of The Distal Knee Joint And Pyarthrosis Following External Fixation*	J Orthop Trauma	12	Autopsy	MRI arthrography plus histopathological dissection	50

In our systematic review of LRR, the mean number of patients per study was 20.2 (range, 9–55 patients), with a mean follow-up of 46 months (range, 1–204 months) (see Table 2). The distal femur was affected in three out of four patients, followed by the soft tissue (11 %), the proximal tibia (7 %), and patella (2 %). Local recurrence rates following extraarticular knee resections for sarcomas varied between 0 and 21 % [3–5, 18, 19]. Hardes et al. [4] and Nakamura et al. [19] routinely resected the STFJ, whereas Anract et al. [18] and Zwolak et al. [5] did not. Capanna et al. [3] reported that the exact extent of resection depended on tumor localization. The analysis of subgroups showed a LRR for “no resection of STFJ” between 0 and 21 %, in contrast to the group including resection of STFJ with LRR between 4 and 8 %.

**Table 2:** This table shows the results of the literature review regarding local recurrence rates after extraarticular knee resection

Author	Journal	Number of patients	Location distal femur	Location proximal tibia	Knee joint	Patella	Follow-up (months)	Time	Resection of STFJ	Local recurrence rate	Details on local recurrence rate
Hardes et al. (2013) Hardes et al. [4]	Bone Joint J	55	51	3	4	1	56 (1–204)	1992–2011	Yes	*n* = 2 (4 %)	2 patients with high- grade osteosarcoma, not treated according to the COSS protocol
Nakamura et al. (2001) Nakamura et al. [19]	Oncol Rep	13	NA	NA	NA	NA	61 (17–45)	1987–1998	Yes	*n* = 1 (8 %)	NA
Zwolak et al. (2011) Zwolak et al. [5]	Clin Orthop Relat Res	11	11	0	0	0	38 (14–80)	2000–2008	No (biceps femoris tendons are kept intact)	*n* = 1 (9 %)	Patient with fibromyxoid sarcoma
Capanna et al. (2010) [3]	Clin Orthop Relat Res	13	4	2	6	1	54 (12–144)	1996–2009	No (depending on the location of the tumor)	*n* = 0–3 (0–21 %)	All recurrences were in the three patients who had marginal or contaminated excisions
Anract et al. (2001) Anract et al. [18]	Clin Orthop Relat Res	9	6	2	1	0	23 (6–30)	1992–1997	No	*n* = 0 (0 %)	NA

*COSS* cooperative osteosarcoma study group, *NA* not available

## Discussion

To clarify whether the STFJ is to be resected in the course of extraarticular knee joint resection in sarcoma patients, we conducted a systematic literature review concerning the frequency of a communication between the TFJ and the STFJ and the reported LRR following extraarticular knee resections for bone or soft tissue sarcomas.

Our review has several limitations. First, only a small number of studies for each research question could be retrieved, and there were no level I or II studies (according to [16]). Second, the follow-up times for LRR as well as tumor entities (soft tissue and bone sarcomas) varied (range 1–204 months), minimizing the comparability of the results. Third, our study question as to whether the STFJ is part of the knee joint was not the primary question of any of the studies investigating LRR.

In contrast to previous studies, Puffer et al. [13] found 100 % communication between the STFJ and the TFJ. This was also the only in vivo study retrieved including weight bearing prior to MR arthrography. Bozkurt et al. [10] had previously shown that the communication rate is higher if histopathological dissection is augmented by MRI. It might be that due to lower intracompartimental pressure in cadavers, which lack synovial fluid, a histopathologist would not see any communication between the STFJ and the TFJ. Instillation of contrast medium in MRI studies compensates for this low intraarticular pressure, even in cadaver studies. Therefore, studies combining histopathological dissection with MRI arthrography might detect increased assess communication rates more accurately [10]. The 100 % communication rate between TFJ and STFJ in the sole in vivo study cannot be ignored, although these results would have to be reproduced in further studies before the STFJ can be considered to be part of the knee.

Again, it is difficult to compare LRR due to the variety of surgical techniques encountered in the studies we identified as relevant. Hardes et al. [4] always resected the STFJ whereas Capanna et al. [3] chose disarticulation of the proximal tibiofibular joint or excision of proximal fibula en bloc depending on the exact location of the tumor. They did not, however, report whether the STFJ was resected in their three patients with LR.

Arguably, tumors involving the STFJ might require extraarticular knee resection if the STFJ is considered to be part of the knee joint. This is a major orthopedic intervention with extensive rehabilitation time and a higher risk for complications [4]. Abdel et al. [20] reported a series of 112 patients with a malignant tumor in the STFJ for whom treatment options were either local extraarticular resection at the STFJ or amputation. There was no case of extraarticular knee resection in that study which, to the best of our knowledge, is the largest published series to date on malignant STFJ tumors [20].

## Conclusions

In conclusion, we found a communication rate between the TFJ and the STFJ of up to 100 %. We feel that despite our small number of studies and low evidence, extraarticular resection without resection of the STFJ should be considered—at least—as contaminated. The influence of the choice of surgical approach on the LRR unfortunately remains unclear. Due to the low number of available studies as well as major differences in study design, current literature does not allow a conclusive interpretation.

## Abbreviations

LR: local recurrence
LRR: local recurrence rate
RCT: randomized controlled trial
STFJ: superior tibiofibular joint
TFJ: tibiofemoral joint
